# Admission systemic inflammation response index is associated with 14-day adverse clinical outcomes in patients with pyogenic liver abscess: a single-center retrospective cohort study

**DOI:** 10.3389/fcimb.2026.1961188

**Published:** 2026-10-29

**Authors:** Jie Zhou, Guanghua Pei, Ling Huang, Li Qi, Minghui Zhang, Ludong Zhao, Fengfeng Zhou, Likun Wang, Shanxin Peng, Ailing Liu, Xidong Li

**Affiliations:** 1 School of Clinical Medicine, Shandong Second Medical University, Weifang, Shandong, China; 2 Department of Infection Control Center, Linyi People's Hospital, Shandong Second Medical University, Linyi, Shandong, China; 3 Infectious Diseases Department, Affiliated Hospital of Shandong Second Medical University, Weifang, Shandong, China; 4 Department of Radiology, Linyi People's Hospital, Shandong Second Medical University, Linyi, Shandong, China; 5 Department of Hepatobiliary Surgery, Linyi People's Hospital, Shandong Second Medical University, Linyi, Shandong, China; 6 Department of Hospital Infection Management, Linyi People's Hospital, Shandong Second Medical University, Linyi, Shandong, China; 7 Department of Laboratory Medicine, Linyi People's Hospital, Shandong Second Medical University, Linyi, Shandong, China

**Keywords:** adverse clinical outcome, inflammatory biomarker, prognosis, pyogenic liver abscess, retrospective cohort study, systemic inflammation response index

## Abstract

**Objective:**

To assess the association between the systemic inflammation response index (SIRI) at admission and 14-day adverse clinical outcomes in patients with pyogenic liver abscess (PLA), and to evaluate its discriminatory performance.

**Methods:**

This single-center retrospective cohort study included 670 adults with PLA admitted to Linyi People’s Hospital between January 2021 and December 2024. SIRI was calculated from the first complete blood count obtained within 24 hours after admission according to the study data-collection criteria. The primary outcome was a composite of adverse clinical events within 14 days. A base logistic regression model and a model additionally including SIRI were fitted in 30 multiply imputed datasets, with complete-case and death-exclusion sensitivity analyses. Restricted cubic splines, receiver operating characteristic curves, and exploratory measures of incremental performance were evaluated.

**Results:**

Of the 670 patients, 100 (14.9%) experienced an adverse clinical outcome, including four deaths. Median SIRI was higher in patients with adverse outcomes [8.95 (4.14-15.64) vs. 5.80 (2.93-11.64); P< 0.001]. In the multiply imputed linear model, the adjusted OR for SIRI was 1.0187 (95% CI, 1.0022-1.0354; P = 0.0262); the complete-case estimate was 1.0163 (95% CI, 0.9994-1.0334; P = 0.0588). After exclusion of four deaths, the adjusted OR was 1.0191 (95% CI, 1.0026-1.0360; P = 0.0233) among 666 patients with 96 nonfatal adverse outcomes. The spline overall association and nonlinearity P values were 0.0557 and 0.4080, respectively. The AUC for SIRI was 0.6072, lower than that for NLR (0.6782; paired DeLong P = 0.0015). Adding SIRI increased the apparent model AUC by 0.0116 (95% bootstrap interval, -0.0006 to 0.0351).

**Conclusion:**

Higher admission SIRI was associated with greater odds of 14-day adverse clinical outcomes in the multiply imputed linear model, but its standalone discrimination was limited. Sensitivity analyses and incremental performance estimates indicate uncertainty, and clinical utility requires further validation.

## 1 Introduction

Pyogenic liver abscess (PLA) is a suppurative infection of the liver caused by bacteria. Common manifestations include fever, abdominal pain, chills, and elevated inflammatory markers; severe disease may progress to sepsis, septic shock, multiorgan dysfunction, and death. Despite advances in diagnosis and treatment, PLA remains a potentially life-threatening infection that requires prompt recognition and management, and adverse outcomes include treatment failure, recurrence, and death (Kuo et al., 2013; Chen et al., 2014; Sohn et al., 2016; Rossi et al., 2022; Wendt et al., 2024; Zhang et al., 2024; Dudina et al., 2026).

Prognostic assessment in PLA currently relies on patient age and comorbidities, hepatic and renal function, microbiologic findings, abscess characteristics, infection severity, and response to treatment. Previous studies have linked septic shock, nutritional status, diabetes mellitus, maximum abscess diameter, and other clinical factors to adverse outcomes (Cho et al., 2017; Xu et al., 2019; Du et al., 2020; Lee et al., 2021). In recent years, composite inflammatory indices derived from peripheral blood cell counts, including the neutrophil-to-lymphocyte ratio (NLR) and platelet-based ratios, have been evaluated as prognostic markers in infectious diseases and PLA (Park et al., 2019; Ko et al., 2022; Li et al., 2022; Zeng et al., 2026).

SIRI integrates neutrophil, monocyte, and lymphocyte counts and may capture both systemic inflammatory activation and immune dysregulation. Higher SIRI has been associated with short- and long-term mortality in sepsis (Ru and Luo, 2023; Xu et al., 2025) and with adverse outcomes in liver-related conditions (Yin et al., 2024; Efgan et al., 2024; Vural et al., 2024). However, evidence regarding its association with the short-term prognosis of PLA remains limited. We therefore examined the association between admission SIRI and 14-day adverse clinical outcomes in a cohort of 670 patients with PLA and compared its discriminatory performance with that of other inflammatory markers.

## 2 Materials and methods

### 2.1 Study design and population

This single-center retrospective cohort study consecutively included patients with PLA admitted to Linyi People’s Hospital between January 2021 and December 2024. Diagnoses were based on clinical manifestations, laboratory findings, imaging, and microbiologic results. Eligibility criteria were applied before duplicate records were removed. After exclusion of 23 ineligible records, 778 records remained; removal of 108 verified duplicate records yielded 670 unique patients. The study was approved by the Ethics Committee of Linyi People’s Hospital (approval no. 202511-H-051), which waived the requirement for written informed consent because deidentified historical medical records were used.

### 2.2 Eligibility criteria

Patients were eligible if they met all of the following criteria:

1. Age ≥18 years;
2. Fulfillment of the diagnostic criteria for PLA;
3. Availability of the first complete blood count obtained within 24 hours after admission and before major therapeutic intervention according to the study data-collection criteria, allowing calculation of SIRI;
4. Availability of complete 14-day clinical outcome data.

Patients were excluded if they had any of the following:

1. An amebic, fungal, tuberculous, or parasitic liver abscess;
2. A concurrent hematologic malignancy;
3. Chemotherapy, radiotherapy, or immunosuppressive therapy within the preceding three months that could substantially affect peripheral blood cell counts;
4. Severely incomplete key study data, including data required to establish eligibility or calculate SIRI;
5. Missing 14-day outcome data.

### 2.3 Diagnostic criteria

The diagnosis of PLA was based on a comprehensive assessment of clinical manifestations, laboratory results, imaging, and microbiologic findings. Patients were required to meet at least one of the following criteria:

1. Abdominal ultrasonography, computed tomography, or magnetic resonance imaging demonstrated an intrahepatic abscess in conjunction with evidence of infection, such as fever, chills, abdominal pain, leukocytosis, or elevated inflammatory markers;
2. Blood or pus culture yielded a bacterial pathogen;
3. Clinical and radiologic improvement after antimicrobial therapy and/or percutaneous aspiration or drainage supported the diagnosis of PLA.

### 2.4 Data collection

Clinical data were extracted from the electronic health record system, including demographic characteristics, comorbidities, clinical manifestations, laboratory measurements, microbiologic results, treatment modalities, and 14-day clinical outcomes. Detailed abscess characteristics, including size, number, location, and morphology, as well as complete information on the timing and adequacy of source control and antimicrobial appropriateness, were not systematically available throughout the cohort and therefore could not be reliably incorporated into the adjusted analyses.

Demographic variables included age, sex, height, and weight. Comorbidities included diabetes mellitus and biliary tract disease. Laboratory variables included white blood cell, neutrophil, lymphocyte, monocyte, and platelet counts; hemoglobin; albumin; alanine aminotransferase (ALT); aspartate aminotransferase (AST); alkaline phosphatase (ALP); serum creatinine; C-reactive protein (CRP); procalcitonin (PCT); international normalized ratio (INR); and prothrombin activity (PTA). Microbiologic variables included blood and pus culture results. Treatment variables included antimicrobial therapy, percutaneous aspiration or catheter drainage, and surgery.

### 2.5 Calculation of SIRI and other inflammatory markers

The primary exposure was SIRI at admission, calculated from the first complete blood count obtained within 24 hours after admission and, according to the study data-collection criteria, before major therapeutic intervention. SIRI was calculated as follows:


SIRI = neutrophil count × monocyte count/lymphocyte count

All cell counts were expressed as ×10^9^/L. Other inflammatory indices were calculated as follows: NLR = neutrophil count/lymphocyte count; platelet-to-lymphocyte ratio (PLR) = platelet count/lymphocyte count; lymphocyte-to-monocyte ratio (LMR) = lymphocyte count/monocyte count; systemic immune-inflammation index (SII) = platelet count × neutrophil count/lymphocyte count; and pan-immune-inflammation value (PIV) = neutrophil count × monocyte count × platelet count/lymphocyte count. One patient had a monocyte count of zero; only LMR was considered unavailable for this patient. Because exact patient-level time stamps for blood sampling and individual outcome events were not consistently available in the retrospective database, strict temporal ordering between biomarker measurement and every qualifying outcome event could not be independently verified for all patients.

### 2.6 Outcome definitions

The primary outcome was the occurrence of at least one adverse clinical event within 14 days after admission. The reanalysis used seven recorded components: all-cause death, transfer to the intensive care unit (ICU), septic shock, escalation of antimicrobial therapy because infection remained uncontrolled, enlargement of an existing abscess, no reduction in abscess size on day 14 compared with admission imaging, and body temperature remaining >38 °C on day 14. Components could overlap, and each patient was counted once for the composite outcome. Once a qualifying event occurred, subsequent improvement did not change outcome classification. For patients discharged without an event, outpatient and readmission records were reviewed through day 14; outcome status was ascertainable for all 670 patients. The original description also included new abscesses and repeat aspiration, drainage, or surgery, but these events were not separately coded and cannot be reliably enumerated from the available data. Their correspondence with the seven recorded components remains unresolved. All-cause mortality was additionally examined as an exploratory outcome.

### 2.7 Statistical analysis

Statistical analyses were performed using R version 4.4.1. Continuous variables are presented as medians with interquartile ranges and categorical variables as counts and percentages. Between-group comparisons used the Wilcoxon rank-sum test and Pearson’s chi-square test. Descriptive analyses used available measurements and report missing counts. CRP, PCT, and AST records reported above or below a measurement limit were approximately represented by their boundary values; analyses restricted to exact measurements were also performed. These exploratory approaches do not recover the unknown true values. These three markers were not included in the regression models.

Records previously excluded solely because CRP or PCT was reported using a measurement-limit qualifier (“<“ or “>“) were re-evaluated during cohort reconstruction. Such observations were no longer treated as patient-level outliers or used as a basis for excluding otherwise eligible patients.

The base logistic regression model included age, sex, admission temperature, diabetes mellitus, sepsis at admission, albumin, INR, and ALP. The extended model additionally included continuous SIRI. Covariates were chosen for clinical relevance and availability at admission rather than by univariable P values. They represented demographic factors, comorbidity, infection severity, and nutritional, coagulation, and hepatobiliary information. Post-baseline variables, including length of hospital stay and in-hospital drainage or surgery, were not included in the adjusted models because they could be influenced by disease course, treatment response, or subsequent clinical events. SIRI components and mathematically related inflammatory indices were not entered simultaneously.

Albumin, INR, and ALP were missing in 5, 59, and 7 patients, respectively; 64 patients had at least one missing model covariate. Under a missing-at-random assumption, chained-equation multiple imputation with predictive mean matching generated 30 datasets with 20 iterations each (seed 20260919). The imputation model included the outcome, SIRI, and the other model variables. Iteration traces, distributions, logged events, and model convergence were examined. Regression estimates were pooled using Rubin’s rules, and nested models were compared using a pooled D1 Wald test. The 606 patients with complete data for both models formed the sensitivity analysis.

As an additional sensitivity analysis, the four patients who died within 14 days were excluded, and the association between SIRI and nonfatal adverse outcomes was reassessed among the remaining 666 patients using the same covariates and multiple-imputation procedure as in the primary analysis.

Restricted cubic splines with three knots at the 10th, 50th, and 90th percentiles of the observed data were used to check continuous predictors individually, leaving the other continuous terms linear. Nonlinear terms were pooled across imputations. For SIRI, an additional joint test assessed the overall association of its two spline terms. The median SIRI was the reference.

ROC AUCs and 95% CIs were estimated using DeLong’s method. Higher SIRI, NLR, PLR, SII, PIV, CRP, and PCT values and lower LMR values were assigned to adverse outcomes. Directions were fixed for this reanalysis and were not reversed after observing an AUC below 0.5. Paired DeLong tests compared SIRI with five other blood cell-derived markers using patients with both measurements; raw and Holm-adjusted P values were reported. CRP and PCT analyses used their own available samples. Youden cutoffs were exploratory.

Apparent AUC, Brier score, continuous net reclassification improvement (NRI), and integrated discrimination improvement (IDI) were calculated within each imputed dataset and averaged over the 30 datasets. Patient-level bootstrap sampling was repeated 1,000 times, with 30 new imputations and refitting of both models in each replicate. Percentile 95% intervals were calculated. This procedure quantified uncertainty in apparent performance; it did not correct optimism or validate the models. Mortality was reported with an exact binomial 95% CI, and SIRI in survivors and decedents was compared using a two-sided exact rank-sum test. No multivariable mortality model was fitted because there were only four deaths. Tests were two-sided with P< 0.05 as a reference threshold; exploratory tests were not comprehensively adjusted for multiplicity.

## 3 Results

### 3.1 Patient selection

Among 801 records initially identified, 23 were excluded according to the eligibility criteria: two aged<18 years, five with a final diagnosis other than PLA, four with hematologic malignancy, two with chemotherapy, radiotherapy, or immunosuppressive treatment within the preceding three months, and ten with severely incomplete key study data. After these exclusions, 778 records remained. A further 108 verified duplicate records were removed, resulting in a final cohort of 670 unique patients. Of these, 100 (14.9%) experienced the 14-day composite adverse outcome, including four deaths, whereas 570 (85.1%) did not (**Figure 1**).

**Figure 1:**
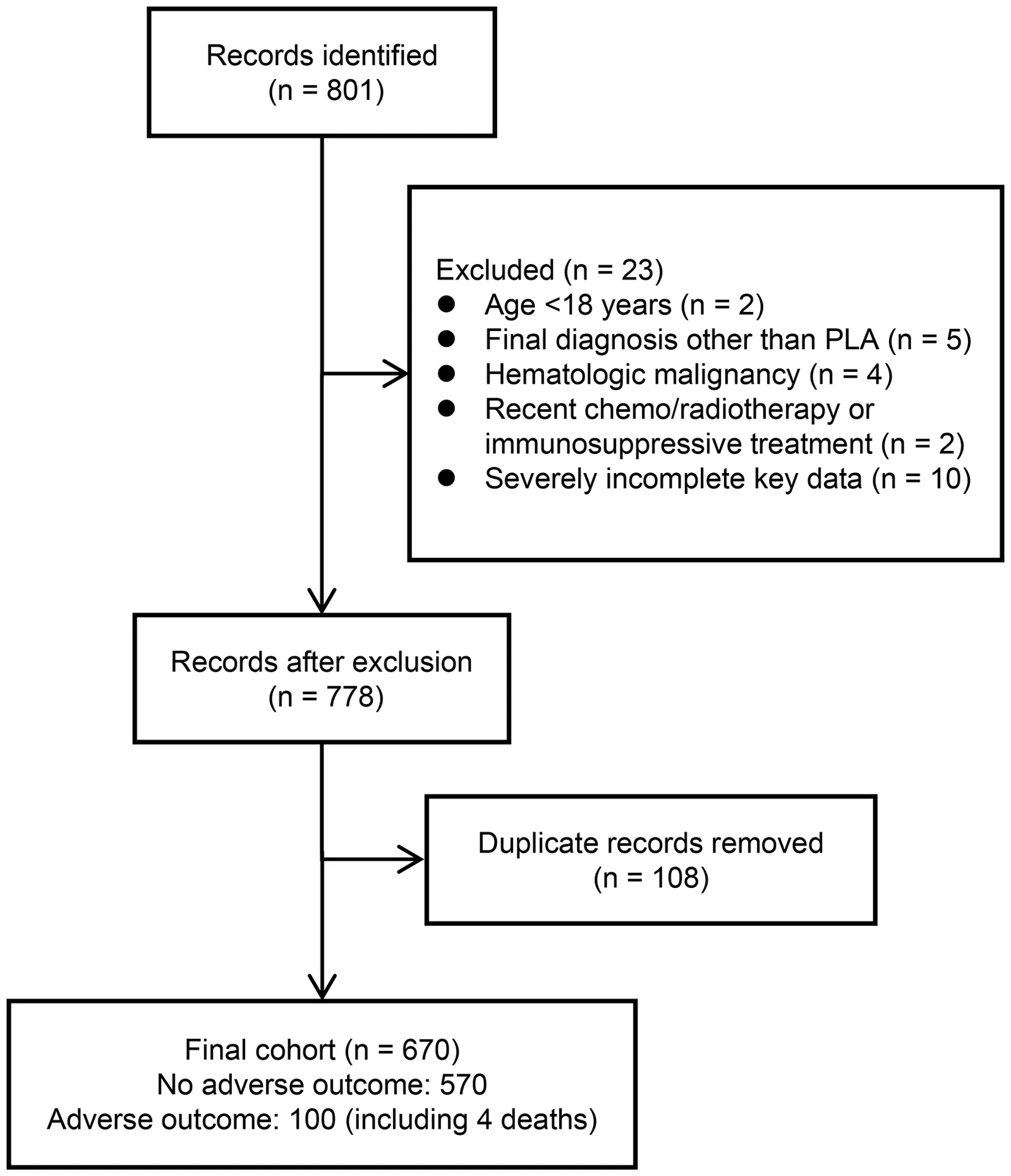
Patient selection flowchart.

The 19 records referred to as “unverified outliers” in the original manuscript were individually traced. Fifteen were retained in the final cohort, and four (record IDs 61, 114, 422, and 481) were removed as duplicate records. All 19 records were therefore reconciled (**Supplementary Table S7**).

### 3.2 Clinical characteristics by 14-day outcome status

Among the 670 patients, 570 had no adverse clinical outcome and 100 experienced an adverse clinical outcome within 14 days. Median age was 62 years, 442 patients (66.0%) were male, and 97 (14.5%) had sepsis at admission. Patients with adverse outcomes had higher SIRI [8.95 (4.14-15.64) vs. 5.80 (2.93-11.64); P< 0.001] and a higher prevalence of sepsis at admission (40.0% vs. 10.0%; P< 0.001). Updated laboratory and demographic comparisons are shown in **Table 1**. CRP, PCT, and AST comparisons were similar in direction under boundary substitution and exact-measurement analyses (**Supplementary Table S5**).

**Table 1:** Clinical characteristics of patients with pyogenic liver abscess.

Variable	Overall N = 670	No adverse outcome n = 570	Adverse outcome n = 100	Missing	P value
Age, years	62.00 (54.00, 70.00)	62.00 (54.00, 70.00)	64.50 (54.00, 71.00)	0	0.294
Weight, kg	66.00 (59.00, 75.00)	67.00 (60.00, 75.00)	65.00 (54.62, 72.12)	38	0.038
Height, cm	167.00 (160.00, 171.00)	167.00 (160.00, 170.00)	165.00 (158.00, 173.00)	59	0.537
Temperature, °C	36.60 (36.40, 37.00)	36.50 (36.40, 37.00)	36.60 (36.40, 37.02)	0	0.782
WBC	11.59 (8.65, 16.09)	11.41 (8.54, 15.25)	13.91 (9.41, 20.42)	0	<0.001
HGB g/L	123.00 (108.25, 134.00)	125.00 (111.00, 135.00)	113.50 (89.75, 130.25)	0	<0.001
RBC 10¹²/L	4.11 (3.65, 4.47)	4.12 (3.71, 4.50)	3.84 (3.16, 4.24)	0	<0.001
PLT	197.00 (123.00, 292.75)	206.00 (132.25, 298.50)	132.00 (47.50, 231.25)	0	<0.001
LYM	1.00 (0.65, 1.48)	1.03 (0.67, 1.54)	0.81 (0.54, 1.20)	0	<0.001
LYM %	8.70 (4.82, 14.30)	9.50 (5.20, 15.20)	5.50 (3.40, 9.50)	0	<0.001
NEU	9.75 (6.82, 14.03)	9.62 (6.68, 13.12)	12.09 (8.07, 18.75)	0	<0.001
NEU %	84.90 (77.25, 90.27)	84.05 (76.20, 89.68)	89.25 (84.30, 92.67)	0	<0.001
MONO	0.65 (0.44, 0.89)	0.66 (0.44, 0.90)	0.60 (0.38, 0.86)	0	0.309
EOS	0.01 (0.00, 0.05)	0.02 (0.00, 0.05)	0.01 (0.00, 0.02)	0	<0.001
BAS	0.02 (0.01, 0.03)	0.02 (0.01, 0.03)	0.01 (0.00, 0.03)	0	0.377
CRP mg/L†	123.90 (68.90, 180.00)	117.00 (64.50, 174.90)	139.90 (85.55, 198.10)	87	0.016
PCT ng/mL†	2.33 (0.44, 14.60)	1.88 (0.42, 10.56)	4.95 (0.83, 25.71)	103	0.002
ALB g/L	31.80 (27.80, 35.80)	32.20 (28.30, 36.45)	28.10 (24.50, 32.45)	5	<0.001
AST U/L†	36.10 (21.58, 70.67)	35.10 (21.20, 66.00)	45.20 (24.00, 155.20)	6	<0.001
ALT U/L	47.75 (24.32, 94.17)	46.40 (23.80, 89.30)	54.00 (26.85, 118.65)	6	0.095
ALP U/L	142.90 (105.00, 219.90)	137.90 (104.00, 208.30)	191.00 (122.67, 296.75)	7	<0.001
Scr μmol/L	65.95 (52.00, 85.00)	65.00 (52.00, 83.00)	66.60 (51.30, 105.50)	8	0.118
INR	1.10 (1.02, 1.20)	1.09 (1.02, 1.18)	1.21 (1.07, 1.32)	59	<0.001
PTA %	85.00 (74.00, 96.00)	86.00 (77.00, 97.50)	73.50 (65.00, 88.25)	59	<0.001
NLR	9.85 (5.40, 18.53)	8.83 (5.09, 16.98)	16.56 (9.04, 26.34)	0	<0.001
PLR	195.63 (124.80, 293.31)	198.17 (131.49, 294.33)	165.34 (63.64, 278.98)	0	0.007
LMR‡	1.62 (1.01, 2.53)	1.64 (1.04, 2.59)	1.46 (0.96, 2.16)	1	0.046
SII	1877.65 (957.67, 3219.08)	1875.82 (1009.30, 3190.27)	1896.35 (720.63, 3507.90)	0	0.664
SIRI	6.14 (3.07, 12.27)	5.80 (2.93, 11.64)	8.95 (4.14, 15.64)	0	<0.001
PIV	1096.97 (512.17, 2254.39)	1125.87 (553.50, 2266.28)	823.64 (292.18, 2201.81)	0	0.119
Male sex	442 (66.0%)	383 (67.2%)	59 (59.0%)	0	0.111
Diabetes mellitus	252 (37.6%)	208 (36.5%)	44 (44.0%)	0	0.153
Biliary tract disease	254 (37.9%)	217 (38.1%)	37 (37.0%)	0	0.839
Sepsis at admission	97 (14.5%)	57 (10.0%)	40 (40.0%)	0	<0.001

Continuous variables are presented as median (interquartile range), and categorical variables as n (%). Groups were compared using the Mann-Whitney U test or Pearson’s chi-square test. WBC, white blood cell count; HGB, hemoglobin; RBC, red blood cell count; PLT, platelet count; LYM, lymphocyte count; LYM%, lymphocyte percentage; NEU, neutrophil count; NEU%, neutrophil percentage; MONO, monocyte count; EOS, eosinophil count; BAS, basophil count; CRP, C-reactive protein; PCT, procalcitonin; ALB, albumin; AST, aspartate aminotransferase; ALT, alanine aminotransferase; ALP, alkaline phosphatase; SCr, serum creatinine; INR, international normalized ratio; PTA, prothrombin activity; NLR, neutrophil-to-lymphocyte ratio; PLR, platelet-to-lymphocyte ratio; LMR, lymphocyte-to-monocyte ratio; SII, systemic immune-inflammation index; SIRI, systemic inflammation response index; PIV, pan-immune-inflammation value. Missing counts refer to unavailable measurements. †Boundary substitution for measurement-limit records; exact-measurement analyses are reported in **Supplementary Table S5**. ‡One LMR value was not calculable because the monocyte count was zero. Cell counts are expressed as ×10⁹/L unless otherwise specified.

The recorded outcome components were death (n = 4), ICU transfer (n = 35), septic shock (n = 27), antimicrobial escalation (n = 51), abscess enlargement (n = 8), no reduction in abscess size on day 14 (n = 2), and temperature remaining >38 °C on day 14 (n = 21). Components overlapped; their union comprised 100 patients (**Supplementary Table S1**). Ninety-nine patients had at least one nonfatal component, including three who also died. By final survival status, there were four deaths and 96 survivors with an adverse outcome. Fourteen-day mortality was 0.60% (exact 95% CI, 0.16%-1.52%). Median SIRI was 14.43 (9.55-18.21) in the four decedents and 6.12 (3.05-12.25) in the 666 survivors (exact P = 0.1409).

### 3.3 Multivariable analysis of SIRI, clinical variables, and 14-day adverse clinical outcomes

The base model included age, sex, admission temperature, diabetes mellitus, sepsis at admission, albumin, INR, and ALP. The extended model additionally included SIRI. Both models converged in all 30 imputed datasets. Higher SIRI was associated with greater odds of an adverse clinical outcome in the extended model (OR per one-unit increase, 1.0187; 95% CI, 1.0022-1.0354; P = 0.0262). Sepsis at admission and INR were positively associated with the outcome, whereas albumin was inversely associated (**Table 2**). The pooled D1 test for adding SIRI gave P = 0.0262. These estimates describe adjusted associations and do not establish clinical predictive benefit.

**Table 2:** Multiply imputed logistic regression models for 14-day adverse clinical outcomes.

Variable and unit	Base model OR (95% CI)	P value	Base + SIRI OR (95% CI)	P value
Age, per year	1.0023 (0.9844 to 1.0206)	0.8023	1.0030 (0.9850 to 1.0214)	0.7422
Male vs. female	0.8733 (0.5303 to 1.4382)	0.5942	0.8337 (0.5047 to 1.3772)	0.4770
Temperature, per °C	1.1661 (0.8385 to 1.6218)	0.3606	1.1782 (0.8480 to 1.6371)	0.3278
Diabetes, yes vs. no	1.0782 (0.6681 to 1.7399)	0.7575	1.0744 (0.6646 to 1.7367)	0.7694
Admission sepsis, yes vs. no	4.2207 (2.4832 to 7.1740)	<0.001	3.9671 (2.3265 to 6.7645)	<0.001
ALB, per g/L	0.9279 (0.8862 to 0.9716)	0.0015	0.9302 (0.8882 to 0.9743)	0.0022
INR, per unit	2.8978 (1.1144 to 7.5353)	0.0292	2.7670 (1.0806 to 7.0851)	0.0339
ALP, per U/L	1.0016 (1.0001 to 1.0031)	0.0367	1.0015 (1.0000 to 1.0030)	0.0512
SIRI, per unit	Not included	—	1.0187 (1.0022 to 1.0354)	0.0262

N = 670 in each of 30 imputed datasets. The base model includes all listed covariates except SIRI; the extended model additionally includes SIRI. ORs are per listed unit, with Rubin-pooled 95% CIs. OR, odds ratio; CI, confidence interval.

### 3.4 Incremental performance of SIRI

The apparent AUC was 0.7515 for the base model and 0.7630 for the extended model. The increase was 0.0116 (95% bootstrap interval, -0.0006 to 0.0351). The Brier score decreased from 0.107021 to 0.106548, a reduction of 0.000473 (95% interval, -0.000363 to 0.004126). Continuous NRI was 0.1603 (95% interval, -0.0488 to 0.3982), and IDI was 0.00535 (95% interval, -0.00066 to 0.03378). All incremental-performance intervals included zero (**Table 3**). The NRI event and nonevent components were -0.3007 and 0.4609, respectively; a positive total NRI did not indicate improvement for both groups. No warnings or imputation events were recorded in the 1,000 bootstrap replicates. Performance was not optimism-corrected.

**Table 3:** Apparent model performance and exploratory incremental value of SIRI.

Marker	MI, N = 670 Estimate	95% bootstrap interval	Complete cases N = 606
Base model AUC	0.751485	0.703744 to 0.809385	0.761615
Base + SIRI AUC	0.763042	0.717845 to 0.818627	0.772295
AUC increase	0.011557	-0.000565 to 0.035138	0.010680
Base model Brier score	0.107021	0.087752 to 0.121869	0.108566
Base + SIRI Brier score	0.106548	0.087288 to 0.120502	0.108379
Brier reduction	0.000473	-0.000363 to 0.004126	0.000187
NRI event component	-0.300667	-0.430451 to 0.322158	-0.340426
NRI nonevent component	0.460936	-0.327603 to 0.569862	0.453125
Continuous NRI	0.160269	-0.048778 to 0.398239	0.112699
IDI	0.005353	-0.000658 to 0.033780	0.003245

Point estimates are averages over 30 imputed datasets. Percentile 95% intervals use 1,000 patient-level bootstrap replicates with repeated imputation and model fitting. Positive Brier reduction denotes a lower Brier score after adding SIRI. These are apparent-performance estimates, not validated performance. NRI, net reclassification improvement; IDI, integrated discrimination improvement.

### 3.5 Sensitivity analyses

The complete-case analysis included 606 patients, of whom 94 had adverse outcomes. The adjusted OR for SIRI was 1.0163 (95% CI, 0.9994-1.0334; P = 0.0588). Its direction and magnitude were similar to the multiply imputed estimate, but the CI included one (**Supplementary Table S2**). The apparent AUC increase was 0.0107 (**Table 3**).

After exclusion of the four patients who died, 96 of the remaining 666 patients experienced a nonfatal adverse outcome and 570 did not. Using the same covariates and multiple-imputation procedure as in the primary analysis, the adjusted OR per one-unit increase in SIRI was 1.0191 (95% CI, 1.0026-1.0360; P = 0.0233), compared with 1.0187 (95% CI, 1.0022-1.0354; P = 0.0262) in the primary analysis. Exclusion of the four deaths did not materially alter the estimated association (**Supplementary Table S6**).

### 3.6 Restricted cubic spline analyses

No continuous predictor showed clear evidence of nonlinearity in the individual spline checks (P values, 0.1662-0.6256; **Supplementary Table S3**). For SIRI, the overall association P value was 0.0557 and the nonlinearity P value was 0.4080. Thus, the spline overall test did not reach the 0.05 threshold, and absence of evidence of nonlinearity did not establish a strictly linear relationship. The two-degree-of-freedom overall spline test differs from the one-degree-of-freedom test in the linear model. **Figure 2** shows the adjusted SIRI curve, with the median (6.14454) as reference and knots at the 10th, 50th, and 90th percentiles.

**Figure 2:**
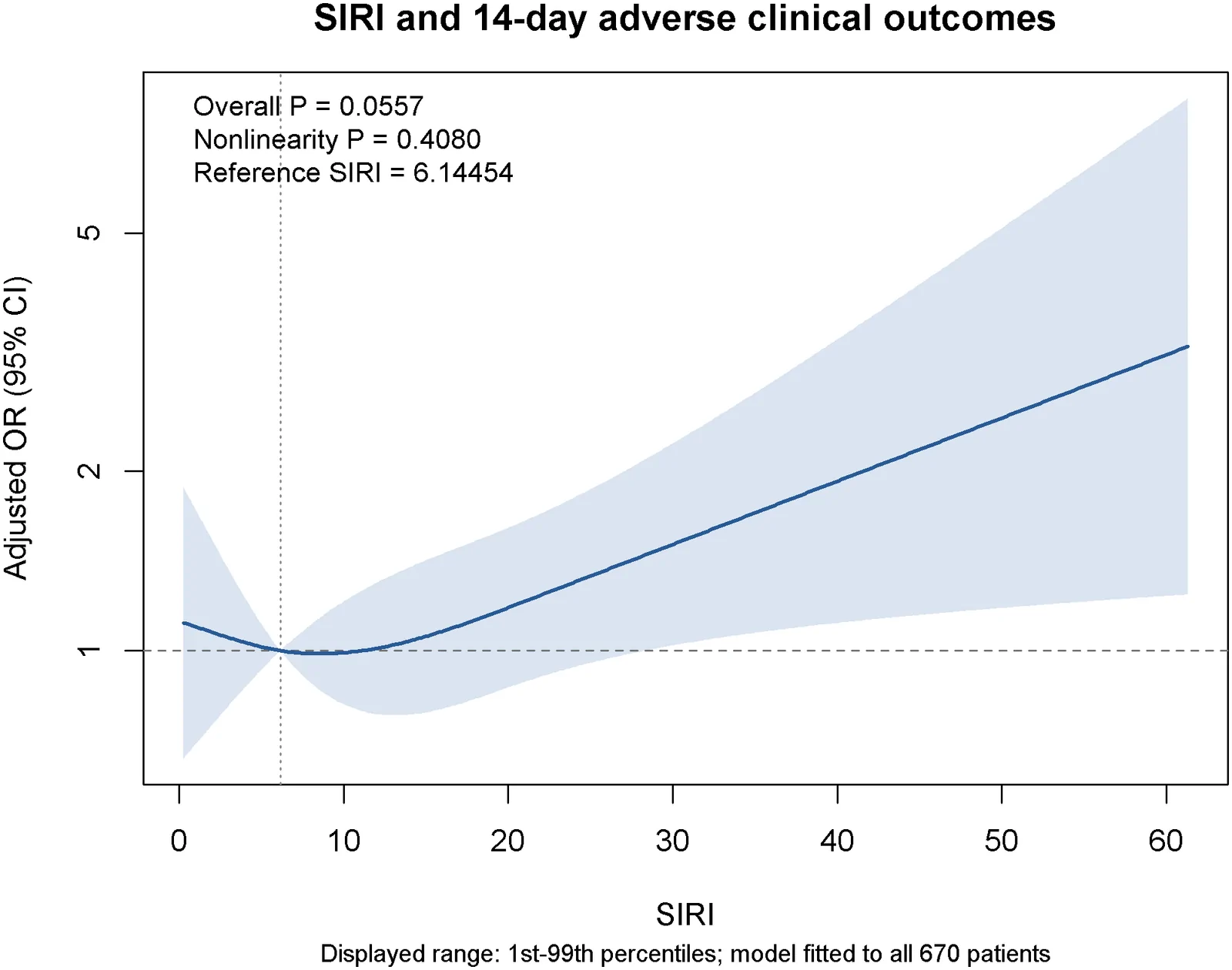
Restricted cubic spline analysis of SIRI and 14-day adverse clinical outcomes. The solid line shows the adjusted OR and shading the 95% CI. The model adjusts for age, sex, admission temperature, diabetes mellitus, sepsis at admission, albumin, INR, and ALP. The reference SIRI is 6.14454. The plot displays the 1st-99th percentiles; all 670 patients contributed to fitting. P for overall association = 0.0557; P for nonlinearity = 0.4080.

### 3.7 Discriminatory performance of inflammatory markers for 14-day adverse clinical outcomes

All individual markers had AUCs below 0.70 in the assigned directions (**Table 4**). NLR had the highest AUC, 0.6782 (95% CI, 0.6256-0.7307), compared with 0.6072 (95% CI, 0.5465-0.6678) for SIRI (**Figure 3**). The paired AUC difference, SIRI minus NLR, was -0.0710 (95% CI, -0.1149 to -0.0272; raw P = 0.0015; Holm-adjusted P = 0.0045). Other paired comparisons are shown in **Supplementary Table S4**. AUCs below 0.5 for PLR, SII, and PIV should be interpreted in light of the fixed scoring directions.

**Table 4:** Discriminatory performance of inflammatory markers for 14-day adverse clinical outcomes.

Marker	N	Events	AUC (DeLong 95% CI)
SIRI	670	100	0.6072 (0.5465 to 0.6678)
NLR	670	100	0.6782 (0.6256 to 0.7307)
PLR	670	100	0.4151 (0.3462 to 0.4839)
LMR	669	100	0.5626 (0.5025 to 0.6227)
SII	670	100	0.4864 (0.4187 to 0.5541)
PIV	670	100	0.4512 (0.3840 to 0.5184)
CRP†	583	90	0.5801 (0.5172 to 0.6430)
PCT†	567	97	0.5977 (0.5343 to 0.6610)

Higher values indicate adverse outcomes except for LMR, for which lower values indicate adverse outcomes. †CRP and PCT use approximate boundary substitution; see **Supplementary Table S5**. AUCs use each marker’s available sample and should not be compared across unequal samples without a paired analysis.

**Figure 3:**
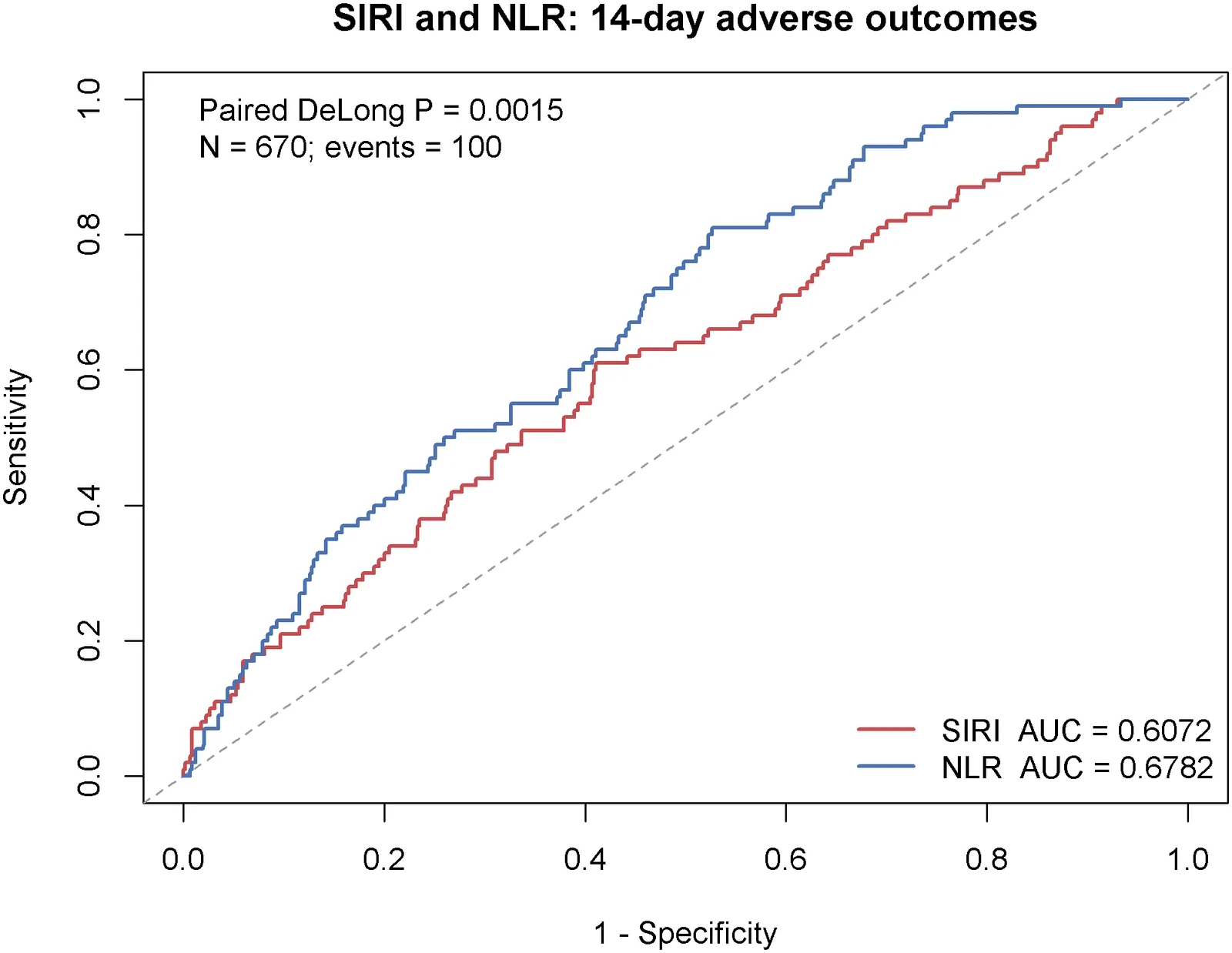
Receiver operating characteristic curves for SIRI and NLR and 14-day adverse clinical outcomes. N = 670; 100 adverse outcomes. Higher values indicate greater risk for both markers. Paired DeLong P = 0.0015.

The sample-derived Youden cutoffs were 7.3842 for SIRI (sensitivity, 61.0%; specificity, 58.9%) and 8.4159 for NLR (sensitivity, 81.0%; specificity, 47.4%). These exploratory cutoffs are not established clinical decision thresholds. Boundary-substitution AUCs for CRP and PCT were 0.5801 and 0.5977; exact-measurement AUCs were 0.5751 and 0.5872. These analyses used different available samples and were not directly compared with SIRI by a paired test. PCT was missing in 100/570 patients without adverse outcomes and 3/100 with adverse outcomes, limiting interpretation.

## 4 Discussion

In this single-center retrospective cohort of 670 patients with PLA, higher SIRI at admission was associated with greater odds of 14-day adverse clinical outcomes in the multiply imputed linear model. However, the complete-case CI included one, and the overall spline test did not reach statistical significance. SIRI alone showed limited discriminatory performance, and the incremental-performance intervals included zero. These findings support further study of SIRI as an associated inflammatory marker, but do not establish its clinical predictive utility.

Previous studies have linked adverse outcomes in PLA to comorbidities, microbiologic findings, abscess characteristics, infection severity, and treatment response (Kuo et al., 2013; Sohn et al., 2016; Cho et al., 2017; Xu et al., 2019; Du et al., 2020; Lee et al., 2021; Rossi et al., 2022; Wendt et al., 2024; Zhang et al., 2024). Rossi et al. (2022) evaluated predictors of primary treatment failure, recurrence, and death; Lee et al. (2021) reported that maximum abscess diameter was independently associated with prolonged hospitalization and poor prognosis; and Du et al. (2020) found that diabetes mellitus influenced short-term outcomes. Peripheral blood cell-derived indices, including NLR and platelet-based ratios, have also been evaluated for prognostic assessment in PLA (Park et al., 2019; Ko et al., 2022; Li et al., 2022). In our study, SIRI was associated with the outcome in the multiply imputed linear model, whereas NLR had a higher standalone AUC than SIRI in a paired comparison. This distinction underscores that statistical association and discriminatory performance are not equivalent. Our findings therefore do not establish the superiority of SIRI over NLR or other markers; whether SIRI provides useful complementary information requires further evaluation.

SIRI combines neutrophil, monocyte, and lymphocyte counts (Ru and Luo, 2023). Neutrophilia may reflect an intensified acute bacterial inflammatory response; monocytes contribute to pathogen clearance, inflammatory mediator release, and tissue repair; and lymphopenia may reflect stress-related immune suppression. The combination of heightened inflammation and impaired immune regulation may contribute to inadequate infection control, progression to sepsis, treatment failure, or death in patients with PLA. Consistent with this interpretation, SIRI has been associated with short- and long-term mortality in sepsis (Ru and Luo, 2023; Xu et al., 2025) and with inflammatory burden and adverse outcomes in liver-related conditions (Yin et al., 2024). These mechanisms remain hypothetical in the context of PLA and require further investigation.

SIRI is derived from routine complete blood count measurements and is inexpensive, readily available, and easy to calculate. However, its low standalone AUC and uncertain incremental performance do not support using it alone to guide treatment decisions. A significant regression coefficient is not equivalent to improved prediction. In particular, the positive total continuous NRI was driven by the nonevent component, while the event component was negative; it cannot be interpreted as 16% of patients being correctly reclassified. Further evaluation should integrate clinical status, imaging findings, microbiologic results, organ function, and response to therapy.

The composite outcome was mainly composed of nonfatal events with differing clinical severity, including persistent fever and lack of imaging improvement on day 14. Antimicrobial escalation and ICU transfer also depend partly on clinical decisions. Exclusion of the four deaths did not materially alter the adjusted SIRI association, but this sensitivity analysis does not identify which individual nonfatal component drives the association. With only four deaths, the exploratory mortality comparison cannot establish or exclude an association with death. The composite-outcome results should not be interpreted as evidence of mortality prediction.

## 5 Limitations

This study has several limitations. First, its single-center retrospective design is susceptible to selection bias, information bias, residual confounding, and limited generalizability. Second, although SIRI was derived from the first blood count obtained within 24 hours after admission and before major therapeutic intervention according to the study data-collection criteria, exact patient-level sampling and event time stamps were not consistently available. Therefore, strict temporal ordering between SIRI measurement and every qualifying outcome event could not be verified for all patients, and dynamic changes in SIRI could not be assessed. Third, the composite outcome included heterogeneous clinical events and depended partly on documentation and treatment decisions. New abscesses and repeat interventions were not separately coded, leaving their correspondence with the recorded components unresolved. Record-based follow-up may also be affected by incomplete documentation, including missed events outside the available hospital records, and the small number of deaths limited mortality-specific analyses. Fourth, although the revised models excluded post-baseline length of stay and treatment variables and incorporated sepsis at admission as an available indicator of baseline infection severity, several potentially important determinants of PLA outcome could not be comprehensively adjusted for. These included detailed abscess size, number, location and morphology, organ dysfunction, microbiologic characteristics, the timing and adequacy of source control, and antimicrobial appropriateness. Residual confounding by disease severity and treatment-related factors therefore remains possible. Fifth, multiple imputation depends on assumptions regarding missingness and model specification; complete-case and measurement-limit sensitivity analyses do not eliminate these uncertainties. Finally, multiple exploratory analyses may produce chance findings, and model performance was apparent, without calibration assessment, optimism correction, or external validation. Prospective multicenter studies are needed to further evaluate the clinical utility of SIRI.

## 6 Conclusion

Among 670 patients with PLA, higher admission SIRI was associated with greater odds of 14-day adverse clinical outcomes in the multiply imputed linear model. The association remained similar after exclusion of the four deaths, although complete-case and spline analyses indicated uncertainty. Its standalone discriminatory performance was limited and lower than that of NLR. Adding SIRI to the base clinical model did not show clear incremental improvement. Its clinical utility, including any role in mortality prediction, requires further investigation.

## Data Availability

The data analyzed in this study contain potentially identifiable clinical information and are not publicly available because of ethical and privacy restrictions. Deidentified data may be available from the corresponding author upon reasonable request, subject to ethics approval and institutional authorization.
