# Supplementary material for: Admission systemic inflammation response index is associated with 14-day adverse clinical outcomes in patients with pyogenic liver abscess: a single-center retrospective cohort study
Source: Front Cell Infect Microbiol. 2026 Oct 29;16:1961188. doi: 10.3389/fcimb.2026.1961188 (PMC13754705; doi:10.3389/fcimb.2026.1961188)
Supplement: Supplementary file 1 [file DataSheet1.pdf]

Supplementary material

Supplementary Table S1. Components of the 14-day composite outcome

Outcome component	n	% of 670	% of 100
All-cause death	4	0.60%	4.0%
ICU transfer	35	5.22%	35.0%
Septic shock	27	4.03%	27.0%
Antimicrobial escalation	51	7.61%	51.0%
Abscess enlargement	8	1.19%	8.0%
No abscess shrinkage on day 14	2	0.30%	2.0%
Temperature >38°C on day 14	21	3.13%	21.0%
Union of seven components	100	14.93%	100.0%

Components overlap. Their union is 100 patients. There were 99 patients with a nonfatal component, including three who died; four deaths and 96 surviving patients with adverse outcomes are mutually exclusive final-status categories. New abscesses and repeat interventions were not separately coded. The death-exclusion sensitivity analysis excludes all four deaths and uses 96 events among 666 patients; the count of 99 overlapping nonfatal-component cases is not its event count (Supplementary Table S6).

Supplementary Table S2. Complete-case logistic regression models

Variable	Base model OR (95% CI)	P value	Base + SIRI OR (95% CI)	P value
Age	1.00676 (0.98779 to 1.02609)	0.4876	1.00744 (0.98843 to 1.02682)	0.4455
Male sex	0.99226 (0.58867 to 1.67257)	0.9767	0.94367 (0.55790 to 1.59618)	0.8288
Temperature	1.12380 (0.79723 to 1.58415)	0.5052	1.13977 (0.80960 to 1.60460)	0.4535
Diabetes mellitus	1.00074 (0.60734 to 1.64896)	0.9977	1.00104 (0.60682 to 1.65135)	0.9968
Sepsis at admission	4.74696 (2.74190 to 8.21825)	<0.001	4.47429 (2.57711 to 7.76810)	<0.001
ALB	0.92290 (0.87894 to 0.96905)	0.0013	0.92486 (0.88065 to 0.97129)	0.0018
INR	2.98386 (1.16120 to 7.66740)	0.0232	2.85722 (1.12436 to 7.26076)	0.0274
ALP	1.00123 (0.99964 to 1.00282)	0.1295	1.00115 (0.99955 to 1.00275)	0.1602
SIRI	Not included	—	1.01627 (0.99940 to 1.03342)	0.0588

N = 606; 94 adverse outcomes. Model covariates are identical to those in Table 2. ORs and Wald 95% CIs are reported.

Supplementary Table S3. Continuous-predictor nonlinearity checks

Variable	Knot at 10th percentile	Knot at 50th percentile	Knot at 90th percentile	Base model Nonlinearity P	Base + SIRI Nonlinearity P
Age	43.00	62.00	77.10	0.530750	0.602700
Temperature	36.20	36.60	37.60	0.166175	0.213415
ALB	24.60	31.80	39.46	0.611008	0.500757
INR	0.970	1.100	1.340	0.324067	0.401291
ALP	81.00	142.90	311.64	0.551075	0.625562
SIRI	1.56522	6.14454	20.9441	—	0.408042

Three knots were fixed at observed-data percentiles. Each variable was expanded separately within 30 imputed datasets. The SIRI overall association P value was 0.055727; the nonlinearity P value was 0.408042.

Supplementary Table S4. Paired DeLong comparisons with SIRI

Comparison	N	AUC difference	95% CI of difference	Raw P	Holm P
SIRI - NLR	670	-0.071026	-0.114881 to -0.027172	0.001502	0.004505
SIRI - PLR	670	0.192105	0.095173 to 0.289037	0.000103	0.000410
SIRI - LMR	669	0.043884	0.008211 to 0.079557	0.015906	0.015906
SIRI - SII	670	0.120772	0.044484 to 0.197060	0.001917	0.004505
SIRI - PIV	670	0.155965	0.097172 to 0.214758	<0.000001	<0.000001

Differences are AUC(SIRI) minus AUC(other). Holm adjustment covers five comparisons. SIRI AUC in the 669-patient comparison with LMR is 0.606467; other comparisons include 670 patients. Fixed directions were retained.

Supplementary Table S5. Measurement-limit audit and sensitivity analyses

Marker	Exact values	Below limit	Above limit	Missing	Available after substitution
CRP	557	4	22	87	583
PCT	542	4	21	103	567
AST	663	0	1	6	664

Marker and handling	No adverse outcome Median (Q1, Q3)	Adverse outcome Median (Q1, Q3)	P value
CRP Boundary substitution	117.00 (64.50, 174.90)	139.90 (85.55, 198.10)	0.01561
CRP Exact values only	113.65 (64.43, 171.15)	137.00 (84.40, 195.75)	0.02891
PCT Boundary substitution	1.88 (0.42, 10.56)	4.95 (0.83, 25.71)	0.00244
PCT Exact values only	1.68 (0.41, 9.16)	4.29 (0.75, 21.59)	0.00862
AST Boundary substitution	35.10 (21.20, 66.00)	45.20 (24.00, 155.20)	0.00092
AST Exact values only	35.10 (21.20, 66.00)	45.20 (23.65, 151.35)	0.00151

Marker and handling	N	Events	AUC (95% CI)
CRP Boundary substitution	583	90	0.580088 (0.517162 to 0.643014)
CRP Exact values only	557	83	0.575123 (0.509977 to 0.640269)
PCT Boundary substitution	567	97	0.597653 (0.534346 to 0.660960)
PCT Exact values only	542	91	0.587230 (0.521370 to 0.653090)

Boundary substitution is approximate. Exact-only analysis excludes measurement-limit records and may also introduce selection bias. Neither approach recovers the true censored measurements. PCT was missing in 100/570 patients without adverse outcomes and 3/100 with adverse outcomes. Confidence intervals do not include uncertainty about the distance beyond a measurement limit.

Supplementary Table S6. Sensitivity analysis after excluding deaths

Analysis	N	Events	Nonevents	Adjusted OR (95% CI)	P value
Primary composite outcome	670	100	570	1.0187 (1.0022-1.0354)	0.0262
Excluding all four deaths	666	96	570	1.0191 (1.0026-1.0360)	0.0233

ORs are per one-unit increase in SIRI. Both analyses adjust for age, sex, admission temperature, diabetes mellitus, sepsis at admission, albumin, INR, and ALP, using the same multiple-imputation procedure. The additional analysis excludes deaths rather than recoding them as nonevents or fitting a separate model for each component. Exclusion of the four deaths did not materially alter the estimated association.

Supplementary Table S7. Disposition of the 19 previously excluded records

Disposition	n	Record identifiers or explanation
Retained in final cohort	15	Included in the 670-patient cohort
Removed as duplicates	4	IDs 61, 114, 422, 481
Total reconciled	19	No record remains of unknown disposition

This audit concerns the 19 records referred to in the original manuscript, rather than all records with a measurement-limit qualifier in the reconstructed dataset. CRP and/or PCT qualifiers were not treated as patient-level outliers or a reason to exclude otherwise eligible patients. The four removed records were duplicates. Record IDs are study record identifiers.
